# Phylogenetic and amino acid conservation analyses of bacterial l-aspartate-α-decarboxylase and of its zymogen-maturation protein reveal a putative interaction domain

**DOI:** 10.1186/s13104-015-1314-6

**Published:** 2015-08-15

**Authors:** Tara N Stuecker, Shanti Bramhacharya, Kelsey M Hodge-Hanson, Garret Suen, Jorge C Escalante-Semerena

**Affiliations:** Department of Bacteriology, University of Wisconsin-Madison, Madison, WI 53706 USA; Microsoft Corporation, 7000 State Highway 161, Irving, TX 75039 USA; Department of Microbiology, University of Georgia, Biological Sciences Building, 120 Cedar Street, Athens, GA 30602 USA; Department of Biological Sciences, University of Arkansas, 850 W. Dickson St., SCEN 601, Fayettevile, AR 72701 USA; Department of Microbiology, University of Georgia, 212C, Biological Sciences Building, 120 Cedar Street, Athens, GA 30602 USA

**Keywords:** Zymogen processing, Pyruvoyl enzymes, Coenzyme A biosynthesis, Pantothenate, Beta-alanine, Aspartate decarboxylase, Pro-PanD maturation, PanM

## Abstract

**Background:**

All organisms must synthesize the enzymatic cofactor coenzyme A (CoA) from the precursor pantothenate. Most bacteria can synthesize pantothenate de novo by the condensation of pantoate and β-alanine. The synthesis of β-alanine is catalyzed by l-aspartate-α-decarboxylase (PanD), a pyruvoyl enzyme that is initially synthesized as a zymogen (pro-PanD). Active PanD is generated by self-cleavage of pro-PanD at Gly24-Ser25 creating the active-site pyruvoyl moiety. In *Salmonella enterica,* this cleavage requires PanM, an acetyl-CoA sensor related to the Gcn5-like *N*-acetyltransferases. PanM does not acetylate pro-PanD, but the recent publication of the three-dimensional crystal structure of the PanM homologue PanZ in complex with the PanD zymogen of *Escherichia coli* provides validation to our predictions and provides a framework in which to further examine the cleavage mechanism. In contrast, PanD from bacteria lacking PanM efficiently cleaved in the absence of PanM in vivo.

**Results:**

Using phylogenetic analyses combined with in vivo phenotypic investigations, we showed that two classes of bacterial l-aspartate-α-decarboxylases exist. This classification is based on their posttranslational activation by self-cleavage of its zymogen. Class I l-aspartate-α-decarboxylase zymogens require the acetyl-CoA sensor PanM to be cleaved into active PanD. This class is found exclusively in the Gammaproteobacteria. Class II l-aspartate-α-decarboxylase zymogens self cleave efficiently in the absence of PanM, and are found in a wide number of bacterial phyla. Several members of the Euryarchaeota and Crenarchaeota also contain Class II l-aspartate-α-decarboxylases. Phylogenetic and amino acid conservation analyses of PanM revealed a conserved region of PanM distinct from conserved regions found in related Gcn5-related acetyltransferase enzymes (Pfam00583). This conserved region represents a putative domain for interactions with l-aspartate-α-decarboxylase zymogens. This work may inform future biochemical and structural studies of pro-PanD-PanM interactions.

**Conclusions:**

Experimental results indicate that *S. enterica* and *C. glutamicum*l-aspartate-α-decarboxylases represent two different classes of homologues of these enzymes. Class I homologues require PanM for activation, while Class II self cleave in the absence of PanM. Computer modeling of conserved amino acids using structure coordinates of PanM and l-aspartate-α-decarboxylase available in the protein data bank (RCSB PDB) revealed a putative site of interactions, which may help generate models to help understand the molecular details of the self-cleavage mechanism of l-aspartate-α-decarboxylases.

**Electronic supplementary material:**

The online version of this article (doi:10.1186/s13104-015-1314-6) contains supplementary material, which is available to authorized users.

## Background

Coenzyme A (CoA) is essential to all forms of life. Prokaryotes use CoA for diverse purposes. In some cases CoA is used as a carrier of naturally occurring or xenobiotic, weak organic acids of varied lengths, while in others CoA is critical to the maintenance of thiol-based redox homeostasis [1–5]. CoA is synthesized from the precursor pantothenate, which can be generated de novo by bacteria, plants and fungi [6]. In bacteria, pantothenate synthesis is a branched pathway in which the intermediates β-alanine and pantoate are generated and then condensed to form pantothenate [7]. l-aspartate-α-decarboxylase (PanD; EC 4.1.1.11) catalyzes the conversion of l-aspartate to β-alanine [8]. l-aspartate-α-decarboxylase is a pyruvoyl enzyme that is synthesized as a 14-kDa zymogen, which undergoes self-catalyzed proteolysis to yield active enzyme [9]. The cleavage reaction yields an 11-kDa α-subunit and 3-kDa β-subunit with the concomitant formation of a pyruvoyl moiety at the *N*-terminus of the α-subunit; the pyruvoyl moiety is necessary for l-aspartate decarboxylation [9].

The *Salmonella enterica*l-aspartate-α-decarboxylase zymogen contains all the determinants needed for maturation. However, at 37 °C, the optimal growth temperature of this bacterium, the ancillary protein PanM (formerly YhhK) is required in vitro and in vivo for cleavage of the l-aspartate-α-decarboxylase zymogen [10, 11]. At present, the mechanism by which PanM stimulates l-aspartate-α-decarboxylase cleavage is not known. What is known is that although PanM is a homologue of Gcn5-like *N*-acetyltransferases (GNATs), it lacks acetyltransferase activity [11]. Interestingly, PanM activity is stimulated by acetyl-CoA, a result that led us to hypothesize that PanM functions as an acetyl-CoA sensor to regulate l-aspartate-α-decarboxylase zymogen maturation [11].

Previously, we showed that the l-aspartate-α-decarboxylase zymogen from *Corynebacterium glutamicum* did not require PanM to process its own maturation [10]. This suggested that two classes of bacterial l-aspartate-α-decarboxylases might exist in nature, one class that would require PanM for processing, and a second class that could mature in the absence of PanM. To determine the distribution of these two forms of PanD in the prokaryotes, we performed a phylogenetic analysis of PanM and l-aspartate-α-decarboxylase. By comparing PanM sequences amongst all homologues, we identified a putative domain of interactions between the l-aspartate-α-decarboxylase zymogen and PanM.

## Results and discussion

### Phylogenetic distribution of prokaryotic l-aspartate-α-decarboxylase and PanM proteins

We determined the distribution of PanM and l-aspartate-α-decarboxylase proteins by searching the National Center for Biotechnology Information (NCBI) database of completed prokaryotic genomes for homologues of the *S. enterica* PanM and l-aspartate-α-decarboxylase proteins. Homologues of the latter were found in 829 genomes (~52 %) (Additional file 1: Dataset S1). Notably, seven l-aspartate-α-decarboxylase homologues were found in members of the Archaea, a finding that differs from those by Genschel who did not find archaeal l-aspartate-α-decarboxylase homologues [12]. Most likely this difference is due to the increase in sequenced genomes since 2004. In contrast, PanM homologues were far less abundant, with only 128 genomes (~8 %) containing homologues of this protein (Additional file 2: Dataset S2). Importantly, all the PanM-containing genomes belonged to the domain Bacteria.

**Fig. 1:**
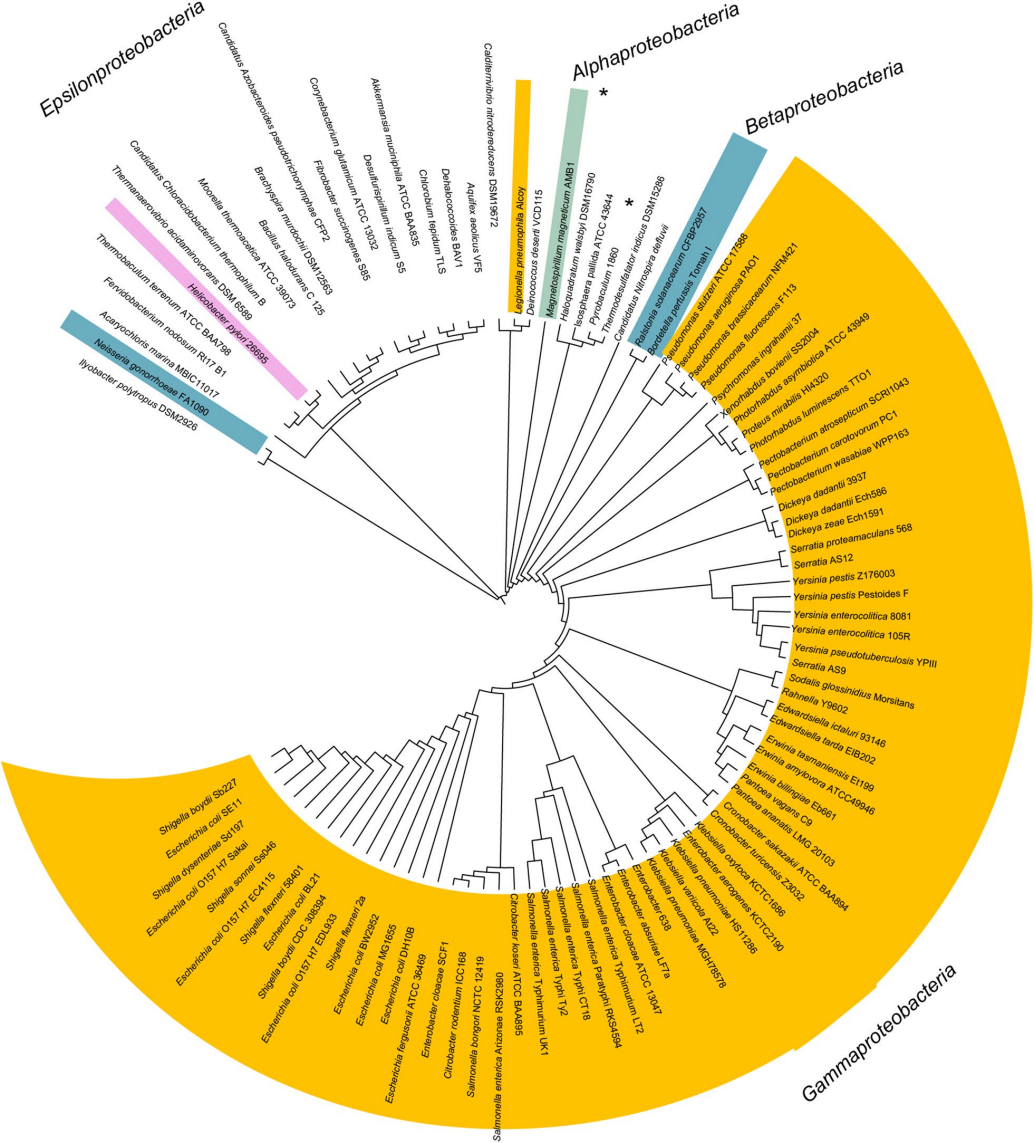
l-Aspartate-α-decarboxylase (PanD) is predominantly found in Gammaproteobacteria. Results of maximum likelihood phylogenetic analysis of l-aspartate-α-decarboxylase homologues in the Proteobacteria are* highlighted*: *teal* Alphaproteobacteria; *blue* Betaproteobacteria; *pink* Epsilonproteobacteria; *yellow* Gammaproteobacteria. Archaeal l-aspartate-α-decarboxylase homologues are marked with an *asterisk*.

**Fig. 2:**
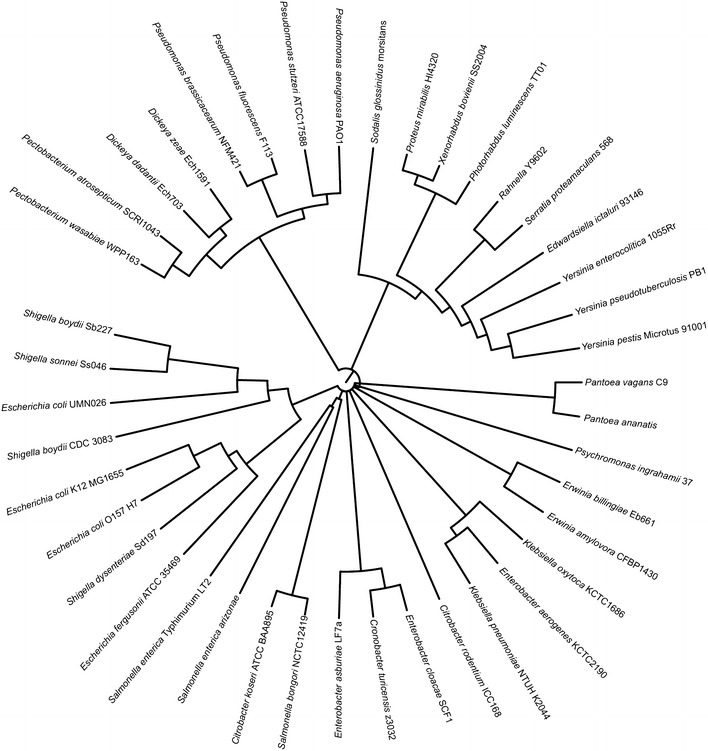
Maximum likelihood tree shows phylogenetic relationships amongst PanM homologues.

To gain insights into the evolutionary history of PanM and l-aspartate-α-decarboxylase, phylogenetic trees were constructed using alignments of all homologues for each protein. l-aspartate-α-decarboxylase was widely distributed amongst numerous bacterial phyla and two archaeal phyla (Fig. 1). Interestingly, the phylogeny of l-aspartate-α-decarboxylase did not follow standard 16S phylogenetic relationships. This was observed in the phylum Proteobacteria (Fig. 1), where l-aspartate-α-decarboxylase homologues from Gamma, Alpha, Beta, and Epsilonproteobacteria did not cluster as expected. In light of this information, we posited that horizontal gene transfer might have played a role in l-aspartate-α-decarboxylase evolution. In contrast, homologues of PanM were only found in genomes belonging to the Gammaproteobacteria (Fig. 2). However, not all gammaproteobacterial genomes contained a *panM* gene. Of the 216 gammaproteobacterial genomes encoding an l-aspartate-α-decarboxylase homologue, only 129 (~60 %) also encoded a PanM homologue. All bacterial genomes encoding a PanM homologue also encoded l-aspartate-α-decarboxylase, which supported the physiological role of PanM as a maturation factor of the l-aspartate-α-decarboxylase zymogen.

We predict that these l-aspartate-α-decarboxylase homologues require PanM for maturation. Notably, ~77 % of genomes encoding l-aspartate-α-decarboxylase lacked a PanM homologue, suggestive of l-aspartate-α-decarboxylase proteins that self-cleave in the absence of PanM.

On the basis of these phylogenetic data we predicted the existence of two classes of l-aspartate-α-decarboxylase enzymes, one class that required PanM for maturation, (Class I), and a second class that did not require PanM (Class II).

### In vivo validation

**Fig. 3:**
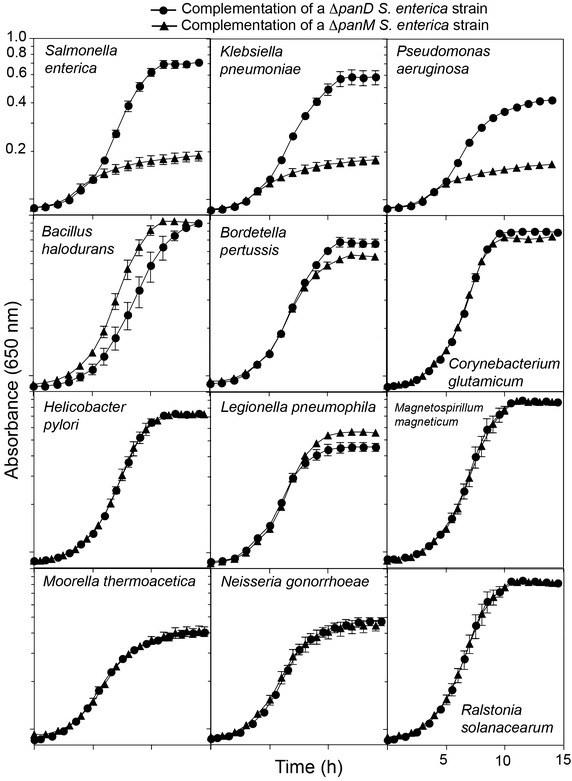
Growth of *S. enterica* Δ*panD* (*circles*) or Δ*panM* strains (*triangles*) on glycerol in the absence of exogenous β-alanine. Each strain expressed the wild-type *panD* allele from the bacterium indicated in the *upper right corner* of each *panel.*

To validate the phylogenetic results, *panD* genes were cloned from several bacteria that contained *panM* and several that lacked *panM.* Each *panD* gene was expressed ectopically in a Δ*panD S. enterica* strain grown on minimal medium devoid of β-alanine to verify l-aspartate-α-decarboxylase function in vivo. To determine whether each l-aspartate-α-decarboxylase could mature in the absence of PanM, the *panD* genes were also expressed in a Δ*panM S. enterica* strain. The *S. entericapanD*^+^ allele was used as control for a *bona fide*l-aspartate-α-decarboxylase that required PanM for maturation [10]. The *C. glutamicum panD*^+^ was included as a control of a gene encoding a l-aspartate-α-decarboxylase that did not require PanM for processing [10] (Fig. 3).

All *panD* homologues restored growth of the *S. enterica* Δ*panD panM*^+^ strain in the absence of pantothenate, indicating that all PanD proteins had l-aspartate-α-decarboxylase activity in vivo (Fig. 3). When expressed in the *S. enterica* Δ*panM* strain, all *panD* homologues from bacteria that also contained a *panM* gene (*S. enterica, Klebsiella pneumoniae, Pseudomonas aeruginosa*) failed to restore growth on minimal medium (Fig. 3, row 1). These data supported the phylogenetic analysis in assigning these l-aspartate-α-decarboxylase homologues to Class I, which we predicted would require PanM for maturation. In contrast, *panD* genes from bacteria that lacked *panM* (e.g., *Bacillus halodurans, Bordetella pertussis, C. glutamicum, Helicobacter pylori, Legionella pneumophila, Magnetospirillum magneticum, Moorella thermoacetica, Neisseria gonorrhoeae* and *Ralstonia solanacearum*) restored growth of an *S. enterica* strain carrying a Δ*panM* deletion in the absence of β-alanine or pantothenate (Fig. 3, rows 2–4). These in vivo results combined with the phylogenetic data supported the existence of two classes of l-aspartate-α-decarboxylase enzymes. Class I l-aspartate-α-decarboxylases that require PanM for activation were present only in the Gammaproteobacteria. Class II l-aspartate-α-decarboxylases that did not require PanM to be active were found in a number of bacterial phyla along with a handful of archaeal species.

### Conserved regions of PanM form a domain where putative interactions with l-aspartate-α-decarboxylases may interact

**Fig. 4:**
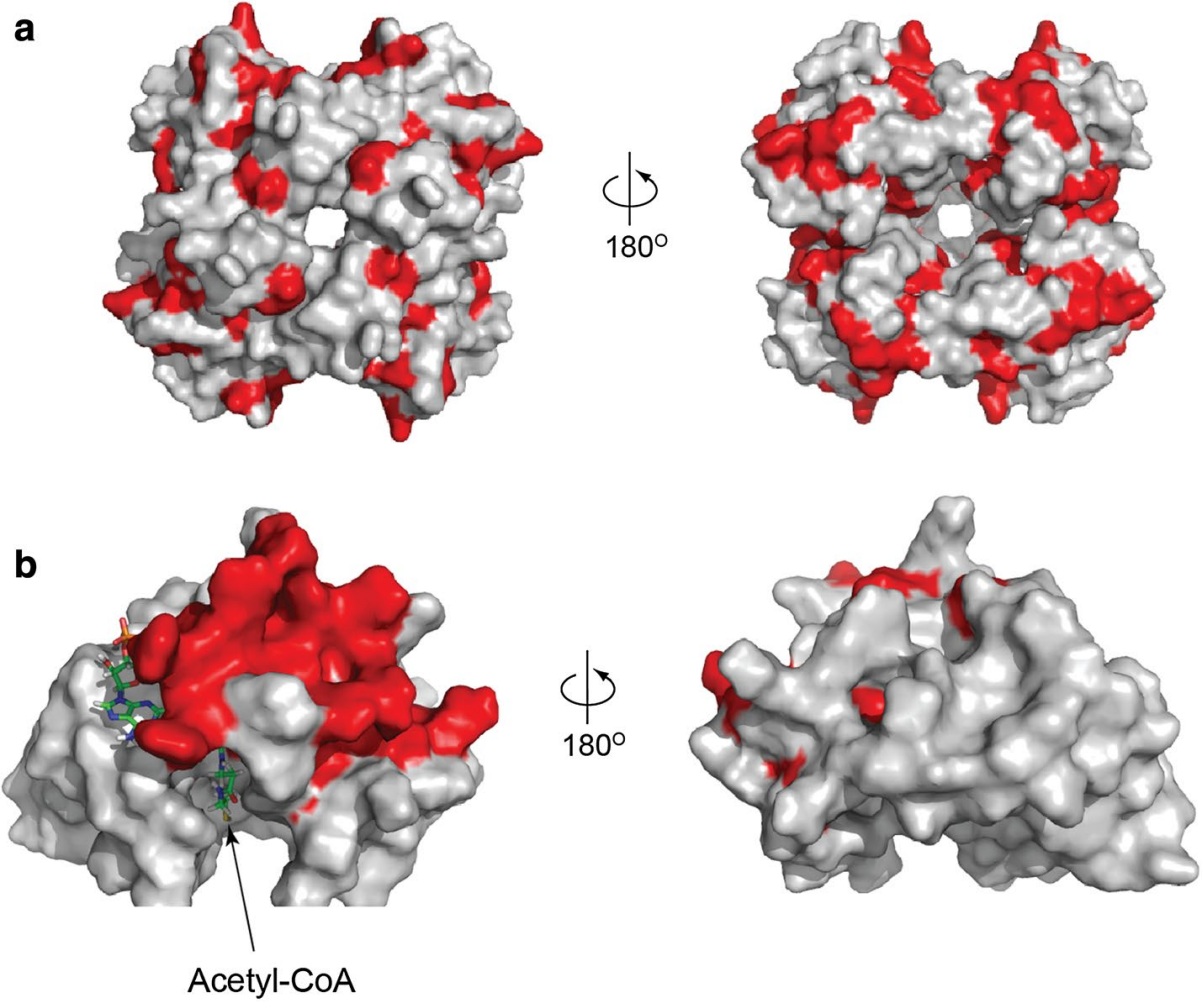
ConSurf analysis of amino acid conservation shows possible interacting surfaces on PanD and PanM. **a** Structure of *E. coli* pro-PanD tetramer. Residues that are more conserved in Class I (PanM-dependent) than in Class II (PanM-independent) gammaproteobacterial PanD homologs are highlighted in *red*. **b** Structure of *E. coli* PanM monomer bound to CoASH with conserved residues highlighted in *red*.

It is known that l-aspartate-α-decarboxylases and PanM interact [10], but the interaction domain has not been identified. We posited that the domain of PanM responsible for interaction with l-aspartate-α-decarboxylase might contain conserved amino acids responsible for the interaction. Given the above data, it followed that l-aspartate-α-decarboxylase residues interacting with PanM should be conserved in the class of homologues requiring PanM for activation (Class I), but not conserved in the class of homologues that did not require PanM (Class II). The ConSurf server [13] was used to calculate evolutionary conservation of amino acids in PanM, and Class I and Class II l-aspartate-α-decarboxylase homologues. Since Class I enzymes were only found in Gammaproteobacteria, we limited our conservation analysis of Class II l-aspartate-α-decarboxylases to the Gammaproteobacteria. Residues that were more conserved in Class I than Class II l-aspartate-α-decarboxylases were highlighted on the crystal structure of *E. coli*l-aspartate-α-decarboxylase zymogen (PDB 1PPY) [14] (Fig. 4a). Residues conserved in all PanM homologues were highlighted on the nuclear magnetic resonance (NMR) solution structure of *E. coli* YhhK (Cort, J. R., Yee, A., Arrowsmith, C. H. and Kennedy, M. A.; unpublished; PDB 2K5T) (Fig. 4b); the *E. coli* YhhK is now known as PanZ [15].

**Fig. 5:**
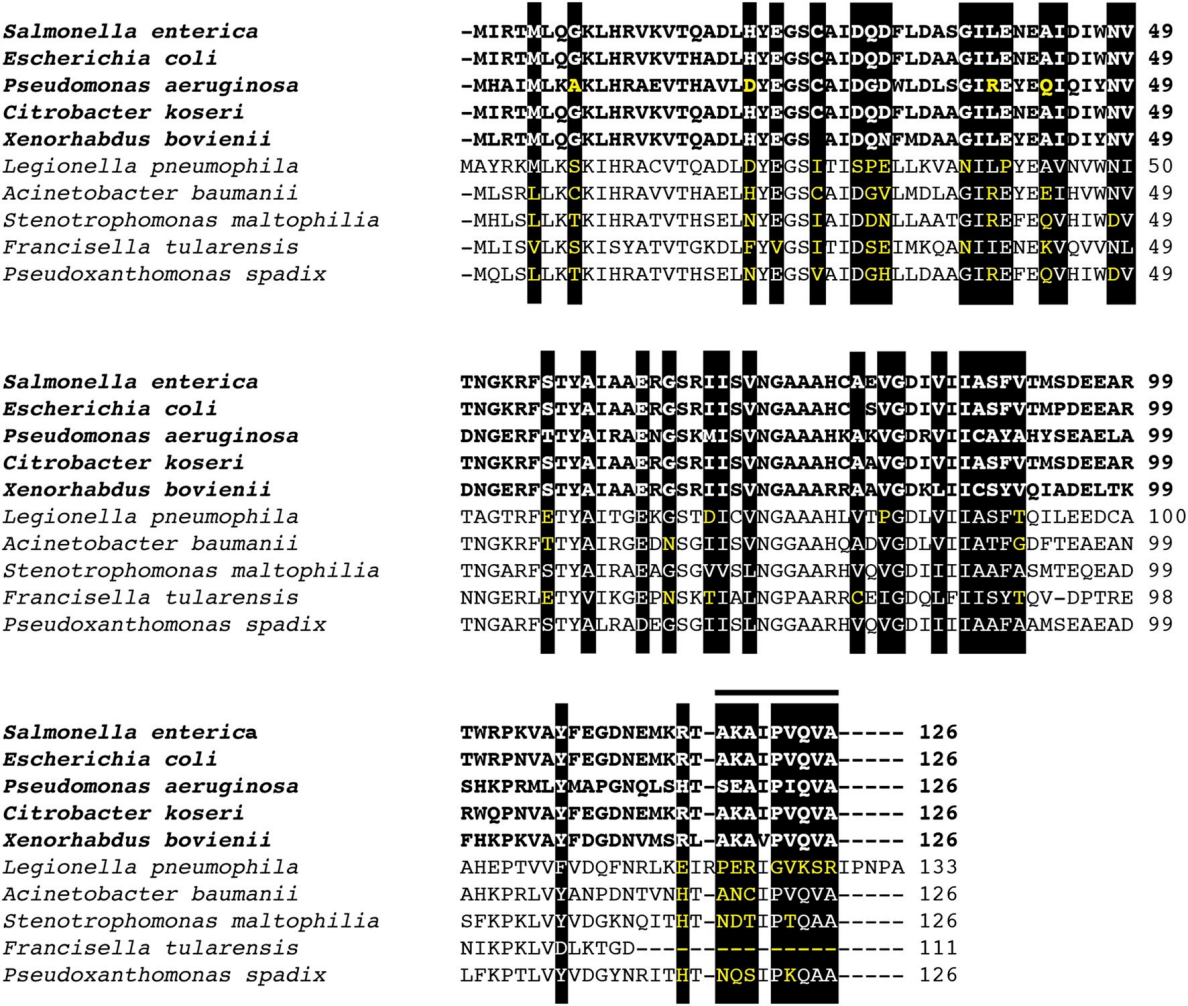
Comparison of PanD protein sequences with residues more conserved in bacteria that synthesize PanM than in bacteria that do not synthesize PanM. Highlighted residues represent potential PanM binding regions. The PanD sequences are shown for five representative bacteria that synthesize PanM (*bold*), and five representative species that do not (*regular text*). Residues that are not conserved are shown in *yellow font*.

**Fig. 6:**
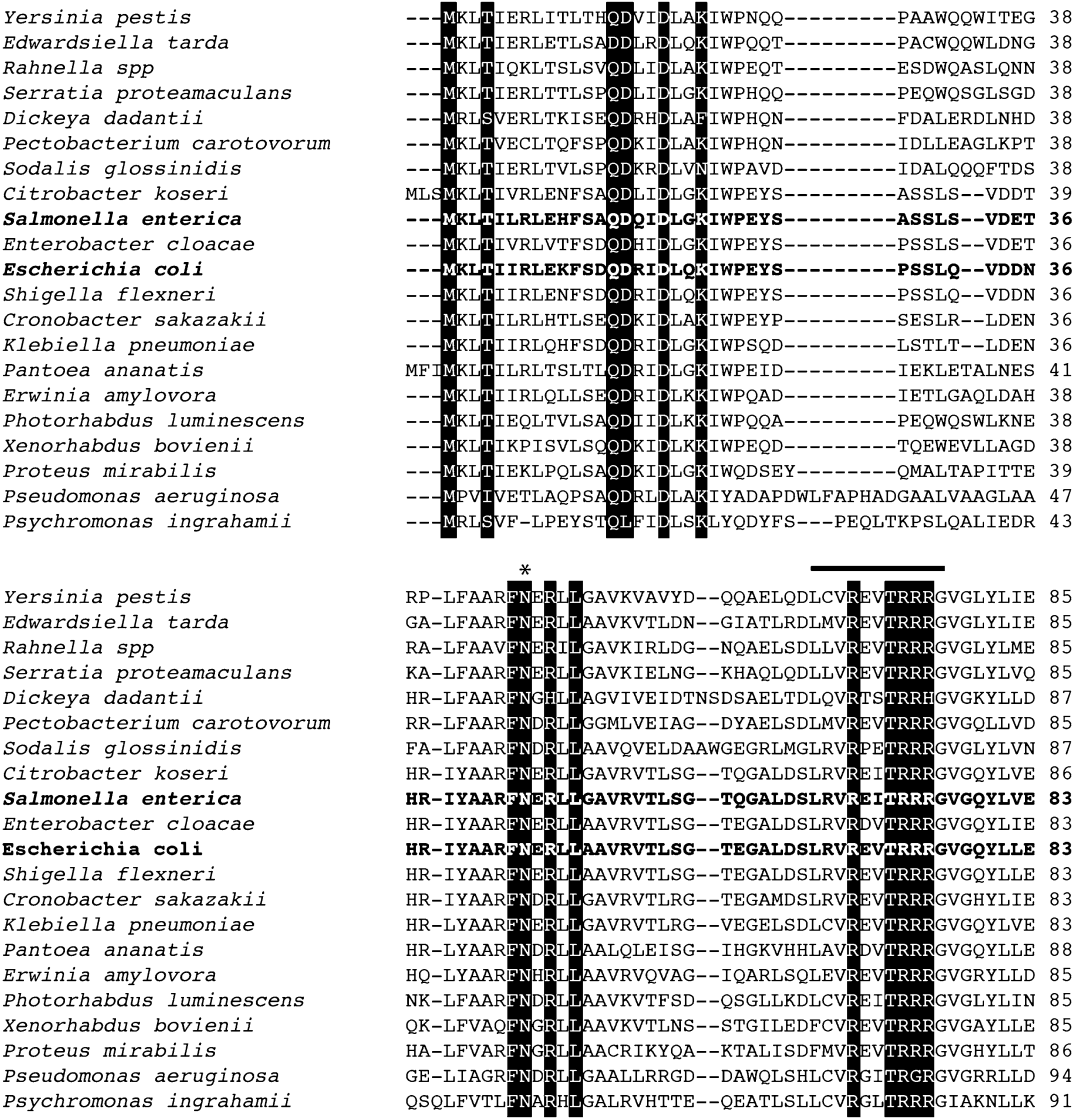
Alignment of PanM proteins used in ConSuf analyses. Surface exposed residues with high conservation highlighted in *black*. These residues form the predicted PanD binding site. The *asterisk* indicates identifies residue Asn45, which was found to be required for PanM binding to pro-PanD [17]. The *bar* atop the sequence on the *lower panel* spans residues Leu66-Gly76, which was also identified by structural studies to be required for PanZ/PanM:PanD interactions [17].

Several conserved regions were present in l-aspartate-α-decarboxylase (Figs. 4a, 5), so further in vitro and in vivo analyses are necessary to determine which conserved region interacts with PanM. However, only one conserved region was observed for PanM (Fig. 4b). Notably, this region was in a unique location when compared to other Gcn5-like *N*-acetyltransferases (GNATs), the family of proteins to which PanM belongs. In GNATs, the protein substrate binds the acetyltransferase in the same cleft to which acetyl-CoA binds [16]. This allows both substrates to be positioned for transfer of the acetyl moiety from acetyl-CoA to the protein substrate. In PanM, the conserved region was located adjacent to the acetyl-CoA binding cleft rather than in the cleft itself (Figs. 4b, 6). If the conserved region of PanM were the PanD interaction domain, PanD would not be positioned for acetyltransfer, supporting previous studies classifying PanM as an acetyl-CoA sensor [11].

During the review of the work reported herein, Monteiro et al. published the structure of *E. coli* PanD in complex with PanM (PanZ in *E. coli*). This elegant and thorough set of studies revealed, among other things, the interaction domain between the PanD zymogen and PanM [17]. Their analysis established that PanM binds to the C-terminus of PanD. In our study, we found several possible PanM binding regions with higher amino acid conservation among organisms that also contained a PanM homologue when compared with those lacking PanM (Fig. 5). Interestingly, the C-terminal portion of PanD identified by Monteiro et al. [17] was one of the possible PanM binding regions identified in our study. Specifically, variations in the motif Ala118 to Ala126 identified by the bar in Fig. 5, may be used to predict which PanD zymogens require PanM to expedite self processing.

For PanM (*E.c.* PanZ), Monteiro et al. also demonstrated that many of the conserved residues found in our study (Fig. 6) were important for PanD binding. Specifically, they found the loop formed by residues Leu66-Gly76 to be stabilized upon Ac-CoA binding, and conserved residue Asn45 on PanM to be critical for PanD binding. Both the Leu66-Gly76 loop and Asn45 were predicted to be involved in PanD binding in our study (Fig. 6). This shows the power of using conservation analysis for the prediction of protein–protein interactions.

There are many intriguing questions regarding the evolution of PanM in pro-PanD maturation. For example, what was the selective pressure that led to the evolution of PanM? This question is interesting in light of the existence of PanM-independent lineages of self-processing pro-PanD proteins.

In *E. coli*, it is known that the PanD zymogen can self-cleave in the absence of PanM. However, maturation, can only occur at high temperatures, optimally at 50 °C [9]. This dependence on high-temperature is not physiological for neither *E. coli* nor *S. enterica*, since neither bacterium can grow at such a temperature. In *E. coli* and *S. enterica*, PanD zymogen maturation in the absence of PanM does not occur, or if it does, the amount of mature PanD generated is insufficient to support growth as indicated by the clear pantothenate phenotype of a *S. enterica panM* or PanZ mutant strain [10, 18].

The availability of the three-dimensional structure of the PanZ/PanM:pro-PanD complex will serve as a critical framework within which we can analyze results from experiments aimed at furthering our understanding of the mechanism of self-cleavage of l-aspartate α-decarboxylases.

## Conclusions

In nature there are two classes of self-cleaving zymogens of l-aspartate-α-decarboxylase (PanD) enzymes. In both classes of zymogens, the cleaving event yields an *N*-terminal pyruvoyl moiety that is critical for substrate binding and catalysis. The putative l-aspartate-α-decarboxylase zymogen:PanM interaction region generated by amino acid conservation analysis using available structural models should facilitate the testing and analysis of the mechanism of l-aspartate-α-decarboxylase zymogen self-cleavage in vivo and in vitro. Structures of l-aspartate-α-decarboxylase zymogens that do not require PanM for maturation would be valuable to better our understanding of the differences between both zymogens and to understand what PanM does to the structure of the zymogen to trigger cleavage.

## Methods

### Phylogenetic analysis

The protein sequences for l-aspartate-α-decarboxylase and PanM were obtained from the complete genome sequence of *Salmonella enterica* serovar Typhimurium LT2 (Accession: NC_003197, accessed: 04/01/2012). Specifically, this corresponded to the GenBank Protein IDs 16763570 (PanD) and 16766851 (PanM). We obtained all protein sequences associated with the complete prokaryotic genome collection found in GenBank (ftp://ncbi.nih.gov/genomes/Bacteria/all.faa.tar.gz, accessed: 04/01/2012) and used the stand alone version of BLAST [19] to format these sequences into a searchable BLAST database. This iteration of the complete prokaryotic genome collection contained a total of 1,606 genome sequences. BLAST was then used to query the l-aspartate-α-decarboxylase and PanM proteins against the complete prokaryotic proteome database and only those alignments with an e value <1e^−03^ were retained.

Phylogenetic trees were then generated as follows. The alignment program MUSCLE [20] was first used to generate an amino acid alignment of all l-aspartate-α-decarboxylase homologues obtained from the complete prokaryotic proteome. This alignment was then imported into the phylogenetic analysis program MEGA [21], and a maximum likelihood (ML) tree was generated. Visualization of this tree was performed using the Interactive Tree of Life web server [22]. For each protein, the originating prokaryotic genus and species was retained throughout the phylogenetic tree construction process. A second tree was also generated using this approach for all homologues of PanM. In both cases, a phylogenetic tree using maximum parsimony was also constructed, but showed no difference in overall topology from the ML tree.

### Plasmid construction

*panD* genes were amplified from genomic DNA listed in Additional file 3: Table S1 using GeneAmp High Fidelity PCR kit (Applied Biosystems) and cloned into the pBAD24 [23] expression plasmid using EcoRI and HindIII restriction sites. Insert sequences were verified using BigDye^®^ sequencing (Applied Biosystems) at the University of Wisconsin Biotechnology Center (Madison, WI, USA). The resulting plasmids are listed in Additional file 4: Table S2.

### Bacterial growth conditions

The Δ*panD* (JE13233) and Δ*panM* (JE12555) *S. enterica* strains [10] were transformed with pBAD24 plasmids expressing *panD* genes (Additional file 4: Table S2). Strains were grown overnight on lysogeny broth (LB) [24, 25] containing 100 μg ml^−1^ ampicillin, then sub-cultured at 0.5 % (v/v) into no-carbon E medium (NCE) [26] containing 20 mM glycerol as the sole source of carbon and energy, and ampicillin (100 μg ml^−1^). Growth was monitored as an increase in optical density at 650 nm using an ELx808 plate reader (BioTek). All growth curves were performed in triplicate and data presented are averaged from at least two independent experiments. Data were graphed using Prism v4.0 (GraphPad).

### Determination of conserved regions in PanD and PanM

PanM protein sequences from bacteria listed in Dataset S2 were aligned using ClustalW2 (http://www.ebi.ac.uk/Tools/msa/clustalW2). For species where multiple strains were listed, only one representative strain was used in the alignment. Conservation of PanM residues was determined from the alignment using ConSurf (consurf.tau.ac.il) [13]. All residues with a ConSurf conservation score above 7 were highlighted on the NMR solution structure of *Escherichia coli* PanM (Cort, J. R., Yee, A., Arrowsmith, C. H. and Kennedy, M. A.; PDB 2K5T) using Pymol [27]. To predict residues of l-aspartate-α-decarboxylase that may participate in interactions with PanM, two alignments of l-aspartate-α-decarboxylase homologues were created. One alignment contained l-aspartate-α-decarboxylase homologues from Gammaproteobacteria that also contained a *panM* gene (Class I). The second alignment contained l-aspartate-α-decarboxylase homologues from Gammaproteobacteria that lacked *panM* (Class II). Both alignments were generated and conserved residues determined as described above. Alignments with conservation scores were manually compared and residues with higher ConSurf scores in Class I compared to Class II l-aspartate-α-decarboxylases were highlighted on the crystal structure of *E. coli*l-aspartate-α-decarboxylase zymogen (PDB 1PPY) [14].

### Availability of supporting data

All data associated with the phylogenetic trees generated in this study are publicly available in the Dryad Digital Repository at http://dx.doi.org/10.5061/dryad.j9d6q.

## Additional files

Additional file 1:
**Dataset S1.** PanD list. Additional file 2:
**Dataset S2.** PanM list. Additional file 3:
**Table S1.** Bacteria from which *panD* genes were amplified and cloned. Additional file 4:
**Table S2.** Strains and plasmids used in this study.
