# Supplementary material for: Phylogenetic and amino acid conservation analyses of bacterial l-aspartate-α-decarboxylase and of its zymogen-maturation protein reveal a putative interaction domain
Source: BMC Res Notes. 2015 Aug 15;8:354. doi: 10.1186/s13104-015-1314-6 (PMC4537548; doi:10.1186/s13104-015-1314-6)
Supplement: Additional file 3: — Table S1. Bacteria from which panD genes were amplified and cloned. [file 13104_2015_1314_MOESM3_ESM.pdf]

Table S1. Bacteria from which *panD* genes were amplified and cloned		
Bacterium	Strain	Source of genomic DNA
Salmonella enterica	LT2	Escalante-Semerena laboratory collection
Corynebacterium glutamicum	ATCC 13,032	H. Holden, University of Wisconsin-Madison
Bacillus halodurans	BAA-125D	American Type Culture Collection
Helicobacter pylori	26695	R. Maier, University of Georgia
Pseudomonas aruginosa	PAO1	P. Greenberg, University of Washington
Klebsiella pneumoniae	M5a1	G. Roberts, University of Wisconsin-Madison
Neisseria gonorrhoeae	FA1090	J. Dillard, University of Wisconsin-Madison
Ralstonia solanacearum	GMI1000	C. Allen, University of Wisconsin-Madison
Moorella thermoacetica	ATCC 39073	American Type Culture Collection
Bordetella pertussis	BAA-589D	American Type Culture Collection
Magnetospirillum magneticum	AMB-1	A. Komeili, University of California-Berkeley
Legionella pneumophila	Lp02	M. Swanson, University of Michigan
