# Supplementary material for: Phylogenetic and amino acid conservation analyses of bacterial l-aspartate-α-decarboxylase and of its zymogen-maturation protein reveal a putative interaction domain
Source: BMC Res Notes. 2015 Aug 15;8:354. doi: 10.1186/s13104-015-1314-6 (PMC4537548; doi:10.1186/s13104-015-1314-6)
Supplement: Additional file 4: — Table S2. Strains and plasmids used in this study. [file 13104_2015_1314_MOESM4_ESM.pdf]

Table S2: Strains and plasmids used in this study.

Strain[a] or plasmid	Genotype or description	Reference
Strains		
**JE12555**	*ara-9 panM751*::*kan*$^{+}$	T.N. Stuecker, K.M. Hodge, and J.C. Escalante-Semerena, Mol. Microbiol. **84:**608-619, 2012
**JE13233**	*ara-9 panD634*::MudJ[b]	T.N. Stuecker, K.M. Hodge, and J.C. Escalante-Semerena, Mol. Microbiol. **84:**608-619, 2012
Plasmids		
**pBAD24**	Expression vector, *bla*$^{+}$	L. Guzman, D. Belin, M. Carson, and J. Beckwith, J. Bacteriol. **177:**4121-4130, 1995
Derivatives of pBAD24		
**pPAN7**	*Salmonella enterica panD*	T.N. Stuecker, K.M. Hodge, and J.C. Escalante-Semerena, Mol. Microbiol. **84:**608-619, 2012
**pPAN14**	*Corynebacterium glutamicum panD*	T.N. Stuecker, K.M. Hodge, and J.C. Escalante-Semerena, Mol. Microbiol. **84:**608-619, 2012
**pPAN16**	*Bacillus halodurans panD*	
**pPAN19**	*Helicobacter pylori panD*	
**pPAN22**	*Pseudomonas aeruginosa panD*	
**pPAN23**	*Klebsiella pneumoniae panD*	
**pPAN25**	*Neisseria gonorrhoeae panD*	
**pPAN36**	*Bordetella pertussis panD*	
**pPAN40**	*Ralstonia solanacearum panD*	
**pPAN41**	*Moorella thermoacetica panD*	
**pPAN56**	*Magnetospirillum magneticum panD*	
**pPAN58**	*Legionella pneumophila panD*	

[a]All strains are derivatives of *Salmonella enterica* LT2

[b]MudJ is an abbreviation of MudI1734 (B.A. Castilho, P. Olfson, and M.J. Casadaban, J. Bacteriol. **158:**488-495, 1984)
